# Monthly Variation of Tetrodotoxin Levels in Pufferfish (*Lagocephalus sceleratus*) Caught from Antalya Bay, Mediterranean Sea

**DOI:** 10.3390/md21100527

**Published:** 2023-10-05

**Authors:** Ali Rıza Kosker, Merve Karakus, Panagiota Katikou, İsmail Dal, Mustafa Durmus, Yılmaz Ucar, Deniz Ayas, Fatih Özogul

**Affiliations:** 1 Department of Seafood Processing Technology, Faculty of Fisheries, Cukurova University, 01330 Adana, Turkey; mdurmus@cu.edu.tr (M.D.); fozogul@cu.edu.tr (F.Ö.); 2 Mediterranean Fisheries Research Production and Training Institute, Demre Unit, 07570 Antalya, Turkey; merve.karakus@tarimorman.gov.tr (M.K.); ismail.dal@tarimorman.gov.tr (İ.D.); 3 Veterinary Research Institute of Thessaloniki, Hellenic Agricultural Organization—DIMITRA, Ktima Thermis, 57001 Thessaloniki, Greece; 4 Vocational School of Aladag, Department of Forestry, Cukurova University, 01720 Adana, Turkey; yucar@cu.edu.tr; 5 Fisheries Faculty, Mersin University, 33169 Mersin, Turkey; ayasdeniz@mersin.edu.tr; 6 Biotechnology Research and Application Center, Cukurova University, 01330 Adana, Turkey

**Keywords:** silver-cheeked toadfish, tetrodotoxin, *Lagocephalus sceleratus*, monthly variation

## Abstract

The silver-cheeked toadfish (*Lagocephalus sceleratus*), an invasive alien pufferfish species that has rapidly settled throughout the Mediterranean region, poses significant threats not only to native marine species and fisheries but also to public health due to the tetrodotoxin (TTX) they harbor. In this study, TTX concentrations in *L. sceleratus* from Antalya Bay in the Northeastern Mediterranean Sea were investigated using Q-TOF-LC-MS on a monthly basis over a one-year period. Pufferfish were caught by angling from May 2018 to April 2019. The TTX levels in three different tissues (gonads, liver, and muscle) of 110 pufferfish in total were determined in both male and female individuals caught for 11 months. The highest TTX mean levels generally occurred in the gonads and the lowest in the muscle samples. As regards the maximum TTX contents, the highest concentrations determined were 68.2, 34.2, and 7.8 µg/g in the gonad, liver, and muscle tissues, respectively. The highest levels were generally observed in late autumn to winter (especially in November and December) in all tissues from both genders. Female individuals were generally found to be more toxic than male individuals. The TTX levels found confirm that the consumption of *L. sceleratus* from Antalya Bay remains dangerous throughout the year, and thus *L. sceleratus* constantly constitutes an important risk source for public health.

## 1. Introduction

Lessepsian species migrating from the Red Sea to the Mediterranean via the Suez Canal are increasing. While some of these alien species are of economic value, species such as pufferfish are considered invasive and poisonous. There are currently eleven established pufferfish species in the Mediterranean [1], among which *Lagocephalus sceleratus* is considered to be the most dangerous and invasive one due to its negative effects on autochthonous species, fishing activities and public health [2]. 

*L. sceleratus* was first reported in 2005 off the coast of Türkiye [3] and then spread rapidly. In a short time, relevant occurrence reports followed from Greece, Croatia, Spain, Libya, Tunisia, Egypt, and Italy [4,5,6,7,8,9,10]. The rapid spread of *L. sceleratus* has negatively affected native species in the Mediterranean ecosystem [11]. A significant part of the ecological success of *L. sceleratus* is due to the fact that it has one of the most advanced types of teeth in the animal kingdom. The ‘first generation teeth’ are covered with recurrent bands of teeth that are continuously renewed by stem cells [12]. These tooth bands fuse to form the upper and lower plates, which together form a beak. This strong beak-like structure allows them to crush and slice even very tough prey organisms such as decapods and bivalves [1,13]. Owing to this unique tooth structure, it can also damage the fishing gear of commercial and recreational fisheries, resulting in significant socio-economic effects on fishermen in affected countries, such as Türkiye [14]. 

In the wider Mediterranean area, pufferfish species have attracted remarkable attention in the last few decades due to their invasion potential as well as the economic damage and poisoning they can cause. For example, the impact of pufferfish on small-scale fisheries in Türkiye is estimated to lead to an annual loss of EUR 2 million (loss of fishing gear and labor) [15]. Similarly, in Cyprus, pufferfish expansion is estimated to generate an annual economic loss of EUR 4173.53 per small-scale fisher. It is also noteworthy that many small-scale commercial fishers in Cyprus have changed their fishing strategies (e.g., using larger nets, fishing for shorter periods of time, and relocating) due to the damage caused by pufferfish to fishing gear [16]. 

In addition to the ecological and economic impacts of pufferfish, the risks posed by *L. sceleratus* to public health are even more worrisome, as this species has also been implicated in poisonings in the Mediterranean, some of which have resulted in patient deaths [17,18,19,20]. This is attributed to tetrodotoxin (TTX), one of the most potent toxins known in the world, which is commonly present in many pufferfish. As such, in the context of public health protection, the EU and many other Mediterranean countries have introduced legislation to ban the placing on the market of fishery products derived from poisonous fish belonging to the families Tetraodontidae, Molidae, Diodontidae, and Canthigasteridae [2,21,22,23].

TTX is a compound with a low molecular weight (319.27) and non-protein structure. It is a water-soluble, colorless, odorless heat-stable toxin. In case of poisoning, symptoms begin to be observed within 2–4 h, and poisoning can result in death because there is no known antidote for this toxin [17]. The origin of TTX is still debated. It is thought to have its origins in bacteria belonging to the *Proteobacteria* phylum, which includes *Pseudomonas*, *Pseudoalteromonas*, and *Vibrio*. However, there are occasional reports of several other bacterial phyla (*Actinobacteria, Bacterioides, Firmicutes,* and *Proteobacteria*) being considered as potential sources of TTX [24,25]. These TTX-producing bacteria, such as *Vibrio, Pseudomonas, Aeromonas, Alteromonas, Nocardiopsis, Bacillus, Shewanella*, and *Roseobacter*, have been discovered in various locations within several aquatic species, including subcutaneous mucus, ovaries, and the gastrointestinal tract [26,27,28,29]. Additionally, some evidence in the literature suggests their association with specific dinoflagellate blooms, such as *Alexandrium tamarense* or *Prorocentrum cordatum* [30,31,32].

TTX and its analogues (TTXs) are found in a taxonomically diverse group of animals, living in both terrestrial and aquatic (marine, freshwater, and brackish) environments; however, pufferfish constitute the most prominent vectors of this toxin [17,33,34]. At the worldwide level, pufferfish are represented by 29 different genera and about 200 different species, and most of these species contain TTX [35]. Some of these pufferfish species that inhabit the Mediterranean also possess TTX at levels that are toxic and potentially deadly [17,36,37,38,39]. To date, most studies on pufferfish toxicity in the Mediterranean have focused particularly on *L. sceleratus* [2,8,33,36,38,39,40,41,42,43,44]. However, there are also scientific studies on the TTX levels of *T. flavimaculosus*, *L. guentheri, L. suezensis*, and *Sphoeroides pachygaster* [2,8,37,45], whereas poisoning cases caused by the consumption of pufferfish have also been reported in different Mediterranean countries [17,46,47]. These poisoning cases mainly stemmed from misidentification and a lack of knowledge on pufferfish. People living in the Mediterranean coastal regions often confuse the pufferfish species encountered in this area with the Japanese pufferfish, which constitute a delicacy (fugu). Nevertheless, it is worth emphasizing that the pufferfish species prevalent in the Mediterranean region do not comply with the classification endorsed by the Japanese Ministry of Health, Labor, and Welfare, designating the pufferfish that are safe for human consumption [48]. 

The studies conducted so far on the toxin content of pufferfish in the Mediterranean region have generally focused on investigating single-sampling or seasonal TTX contents [2,8,33,36,38,39,40,41,42,43,44]. Considering, however, the extremely harmful effects that pufferfish may have on public health, it would also be beneficial to determine the monthly changes in TTX levels of these organisms. Poisoning cases in many countries appear on an occasional basis, indicating the importance of monitoring the variation of TTX levels in pufferfish over time. On the other hand, clinical trials of anti-cancer and anti-pain drugs containing TTX are ongoing [42]. As such, exploitation of *L. sceleratus* for TTX purification could be of help to stabilize their population and reduce their impact on the environment. It would therefore be useful to investigate the variation in TTX levels in pufferfish throughout the year in order to target fishing efforts towards the months when TTX levels are highest. To address these considerations, the present study investigated the monthly and sex-related changes in TTX levels of the population of *L. sceleratus* in Antalya Bay.

## 2. Results and Discussion

Only sexually mature pufferfish were included in this study. The maximum mean weights of the female and male samples were 2062.2 g and 1665.4 g, respectively, while the maximum mean lengths of the females and males were 54.5 cm and 52.5 cm, respectively (Table 1). 

**Figure 1:**
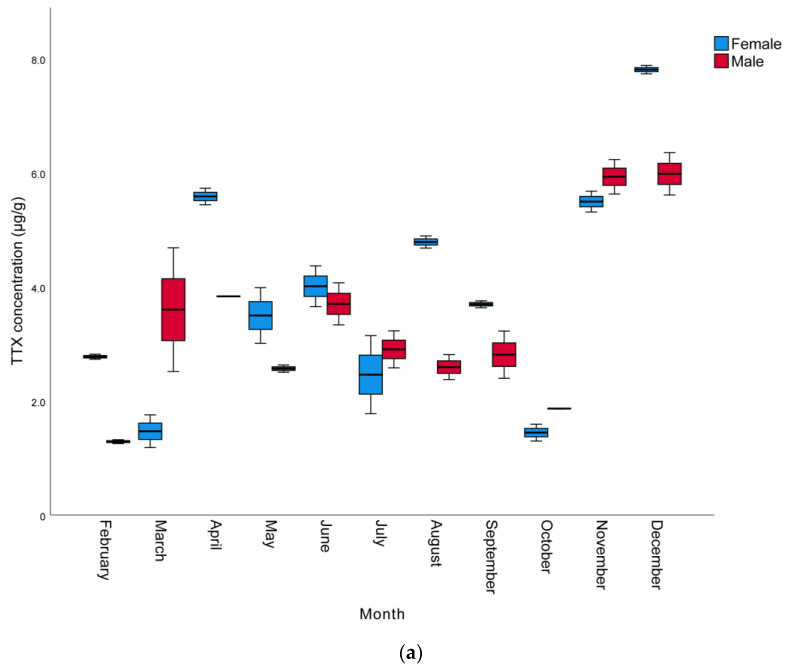

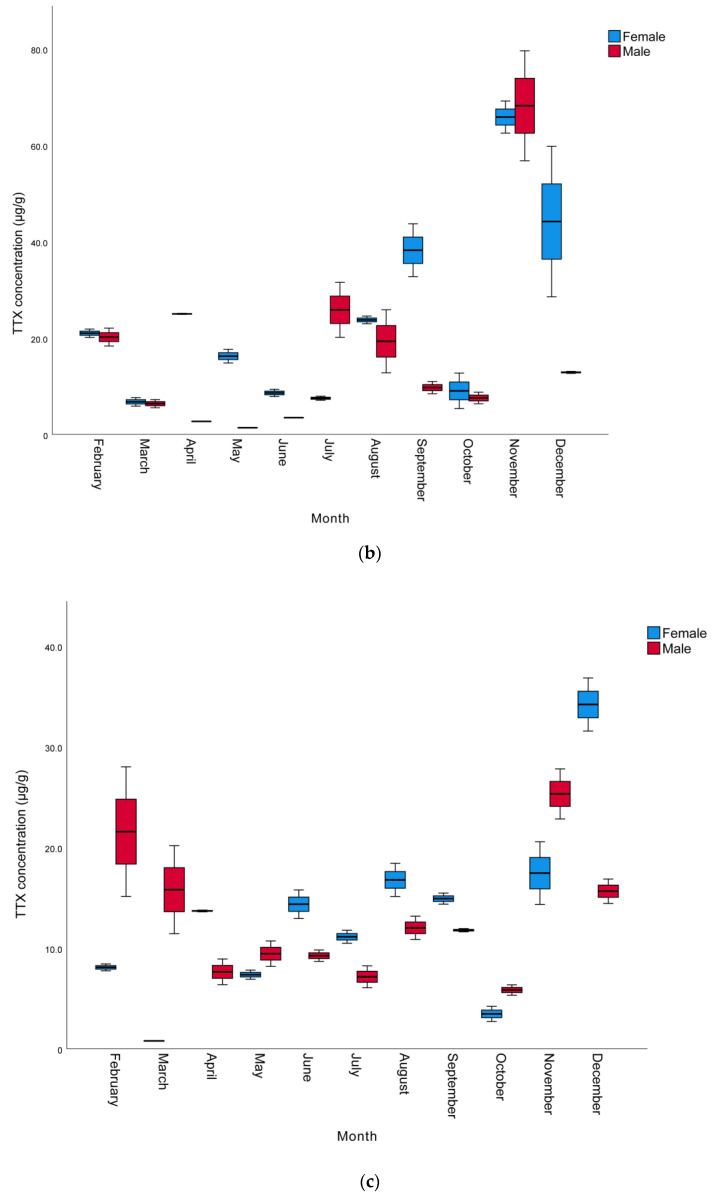
Monthly variation of TTX levels (µg/g) of male and female *L. sceleratus* individuals in (**a**) muscle, (**b**) liver, and (**c**) gonads (*n* = 5, error bars represent SD).

The monthly and sexual variation of TTX levels in the muscle, liver, and gonads of *L. sceleratus* caught in Antalya Bay are presented in Table 2, whereas a graphical comparison of TTX contents in different tissues of male and female individuals is shown in Figure 1. The TTX levels measured were in the range of 0.8 to 68.2 µg/g. The highest TTX mean levels were present in the gonads and the lowest in the muscle samples. The maximum toxin concentrations determined per tissue were 68.2, 34.2, and 7.8 µg TTX/g in the gonad, liver, and muscle, respectively. The highest TTX concentrations for all tissues and both sexes were observed in November, with female individuals generally harboring more toxins than male ones. However, in some months, especially between October and March, liver TTX contents were higher in males than females. In terms of monthly toxin levels, the TTX levels of most female individuals were found to be higher than those of males. 

In terms of the TTX levels measured in the tissues throughout the year, the order was found to be gonad > liver > muscle. This is in agreement with other studies conducted by Christidis et al. [39], Rambla-Alegre et al. [8], Alkassar et al. [49] and Kosker et al. [2,42], where the highest toxin concentrations were also observed in the gonads and the lowest toxin levels were detected in the muscle tissue. The TTX levels in the gonads were within the range of 1.4–68.2 µg/g, similarly to the ranges reported for *L. sceleratus* individuals from the Aegean Sea [33,36,41,50], the Adriatic Sea [51], the Eastern Mediterranean [42,43], the Western Mediterranean [8] and the Southern Mediterranean [39]. The TTX levels in the liver were in the range of 0.78–34.19 µg/g, also in accordance with the levels detected in *L. sceleratus* from the Aegean Sea [36,41], the Eastern Mediterranean [40,42] and the Southern Mediterranean [39]. However, as can be seen in Table 3, the TTX levels in the livers of pufferfish caught in Antalya Bay were higher than the values reported by Reverté et al. from the Aegean Sea [33], Kosker et al. from the North East Mediterranean [2], Rambla-Alegre et al. from the West Mediterranean [8] and Acar et al. from the East Mediterranean [43], while they were lower than those reported in the livers of *L. sceleratus* from the southern Aegean by Anastasiou et al. [50]. Finally, the muscle tissue TTX concentrations (1.3–7.80 µg/g) were equivalent to those reported by Katikou et al. from the Aegean Sea [41], Kosker et al. from the North East Mediterranean [2] and Christidis et al. from the South Mediterranean [39]. On the other hand, the TTX levels determined in the present study in the gonads were lower compared to some other studies [40,43,49,50] conducted nearby. This could be attributed to differences in the sampling period, which in the other studies was only seasonal and probably involved one single sample per season, in contrast to the monthly sampling of the present study. This hypothesis is further supported by the notable monthly fluctuations in TTX concentrations, indicating that the sampling intervals’ periodicity may hold a significant role. However, differences in environmental conditions in the respective sampling periods/years, such as temperature, availability of nutrients, etc., as well as specimens’ sizes and ages, may well account for the variation of toxin levels observed between individual studies.

Considering monthly changes, a statistically significant increase was observed in the TTX levels in all tissues, especially towards the late autumn to early winter months (*p* < 0.05). The mean TTX levels in the muscle, liver, and gonads of male pufferfish in November were 5.9, 25.3 and 68.2 µg/g, respectively. In females, the mean TTX levels in the gonads was 65.9 µg/g in November, while the highest TTX levels in the muscle and liver tissues (7.8 and 34.2 µg/g), respectively, were observed in December. The spawning season of *L. sceleratus* in the Mediterranean includes a very wide period and can last from April to September [1,44,52]. It has been reported that the TTX molecule is a reproductive adaptation for pufferfish and that they show maximum toxicity in the spring and summer seasons, which are the breeding seasons of pufferfish [53,54]. However, Yu and Yu [55] reported that *Takifugu niphobles* and *Takifugu alboplumbeus* pufferfish are not toxic during their spawning period. Similarly, according to the findings of the studies conducted by Katikou et al. [41], Kosker et al. [2,42] and Acar et al. [43], *L. sceleratus* living in the Mediterranean seem to contain higher TTX in the late autumn months after the spawning period. Interestingly, in these cases, pufferfish show higher toxicity outside of their breeding season, a phenomenon that may be specific to the Mediterranean ecosystem. 

Overall, the TTX levels of *L. sceleratus* caught in Antalya Bay are generally in agreement with those reported from different parts of the Mediterranean by means of analytical methods (Table 3). On the other hand, specifically where potential poisoning caused by muscle tissue consumption in humans and wild animals or pets is concerned, almost all of the pufferfish examined in this study were toxic, that is, exceeding the sole existing safety threshold of 10 mouse units (MU) TTX eq/g (equivalent to 2.2 µg TTX eq/g) established by the Japanese authorities [56]. Similarly, high concentrations of TTX have been reported in other Mediterranean pufferfish species, *L. suezensis* and *T. flavimaculosus* [2,37]. However, the main responsible species for poisoning cases in the Mediterranean is *L. sceleratus* [17]. At earlier times, these latter pufferfish were sold at fish stalls, probably due to their larger individual size and their widespread presence in almost all Mediterranean countries [57]. Currently, the trade of pufferfish is restricted by legal regulations in Türkiye, the EU, and other Mediterranean countries [22,23], but still, several poisoning cases resulting from the consumption of *L. sceleratus* have occurred in some Mediterranean countries [17,19,46]. These cases are commonly the result of accidental consumption of pufferfish caught during amateur fishing by people living in coastal areas. Nevertheless, some of the poisoning incidents have been caused by pufferfish caught professionally by fishing lines, especially in the Antalya Bay [17]. 

As aforementioned, *L. sceleratus* is not listed as an edible pufferfish species in Japan [28], mainly due to the fact that its muscle commonly contains TTX at levels surpassing the safety threshold of 2.2 μg TTX eq/g. This limit was established to ensure the safe consumption of pufferfish meat in Japan but has also been employed as a toxicity assessment criterion for *L. sceleratus* samples from the Mediterranean Sea [17,20]. Taking into account this threshold value, the TTX levels found in the present study generally indicate a substantial risk of foodborne poisoning associated with the potential ingestion of *L. sceleratus* edible tissues (muscle, liver, and gonads) caught by angling in Antalya Bay, regardless of the time period of sampling. As such, *L. sceleratus* should continue to be considered as an important source of risk in terms of public health in addition to its ecological and economic consequences. 

It is noteworthy to mention that currently, TTX levels in seafood are a subject of debate, especially in the European Union. The European Food Safety Authority (EFSA) Panel on Contaminants in the Food Chain (CONTAM) has concluded that a concentration of 44 µg TTX/kg of shellfish meat, as regards marine bivalves and gastropods, is not expected to lead to adverse effects in humans [29]. This threshold value is notably more conservative compared to the relevant Japanese safety limit for the assessment of edible pufferfish. Subsequently, newer toxicological data obtained by administering TTX in mice through feeding, instead of gavage, but following the same calculation logic as that of the EFSA Panel, indicated that a higher concentration of 560 µg TTX/kg of shellfish meat would be expected not to lead to adverse effects in humans [58]. In addition, the EFSA opinion has also suggested exploring whether paralytic shellfish poisoning toxins (saxitoxins, STXs) and TTXs should be combined into one health-based guidance value due to their similarities as regards toxic effects and mode of action [29]. In this context, a recent study has concluded that the current regulatory threshold of 800 μg STX eq./kg [22], which is much higher than the EFSA proposed limit for TTXs alone, would be fit for purpose for the combined presence of STXs and TTXs [59]. Nevertheless, regardless of which of the above threshold values is used, almost all the pufferfish tested in our study would have been deemed unsuitable for human consumption.

## 3. Materials and Methods

### 3.1. Tetrodotoxin Standard 

The TTX standard (1 mg powder, purity 99%) used for the instrumental toxin analysis was purchased from Abcam Biochemicals (Cambridge, UK). The TTX standard was diluted with methanol (Merck, Darmstadt, Germany) containing 0.01M acetic acid to obtain the stock TTX standard. Working standard solutions at concentrations of 0.05, 0.1, 0.5, and 2 μg/mL were prepared from the stock solution to obtain a calibration curve (*R*^2^ = 0.9994). The standards were kept at −20 °C until used.

### 3.2. Fish Collection, Measurements, and Identification 

The pufferfish were caught by angling at monthly intervals between May 2018 and April 2019, except for January 2019, where sampling was not possible due to weather conditions and technical difficulties. The fish were transported to the laboratory on ice. Length–weight measurements of the pufferfish caught in all months were obtained (Table 1), and sex determinations were made by examining the gonads of fish with the help of a microscope. A total of 110 pufferfish, 5 male and 5 female, were examined for each month. Only individuals that had reached sexual maturity and sizes likely to be caught by amateur fishermen were used. 

### 3.3. Preparation of Samples and Toxin Extraction

Fish samples were dissected to obtain their dorsal muscle (carefully avoiding the gastrointestinal tract), gonads, and liver. ΤΤΧ extractions in the liver, gonads, and muscle tissues were performed according to the method of Silva et al. [34]. Briefly, 1 g of each tissue was extracted by adding 3 mL of methanol containing 1% acetic acid, and the mixture was homogenized with an Ultra Turrax (IKA T25 Digital Ultra Turrax, Staufen, Germany) at 7200 rpm for 10 min. Then, the homogenized extracts were placed in an ultrasonic bath (Bandelin Sonorex RK 100, Berlin, Germany) at 100 Hz for 10 min. The extracts were kept at room temperature for 15 min and were then centrifuged at 4500 rpm and 4 °C, for 20 min (Hettich Zentrifugen, Universal 32R, Tuttlingen, Germany). The supernatants were collected, and the same procedure was repeated for the pellet residue. The supernatants from both extractions were combined, and the extracts were made up to 7 mL. The extracts were vortex-mixed before proceeding to solid–phase extraction (SPE). After that, 1 mL of the extract was cleaned by running it through a 500 mg/3 mL C18 solid-phase extraction (SPE) cartridge (Supelco, Bellefonte, PA, USA). The sample was eluted with 10 mL of 100% methanol and diluted with the same solvent to a final volume of 12 mL. It was then vortex-mixed and evaporated to dryness using a rotary evaporator. The dry residue was reconstituted with 1 mL of methanol, filtered through 0.45 µm membrane filters, and transferred to vials for analysis.

### 3.4. Tetrodotoxin Analysis

**Figure 2:**
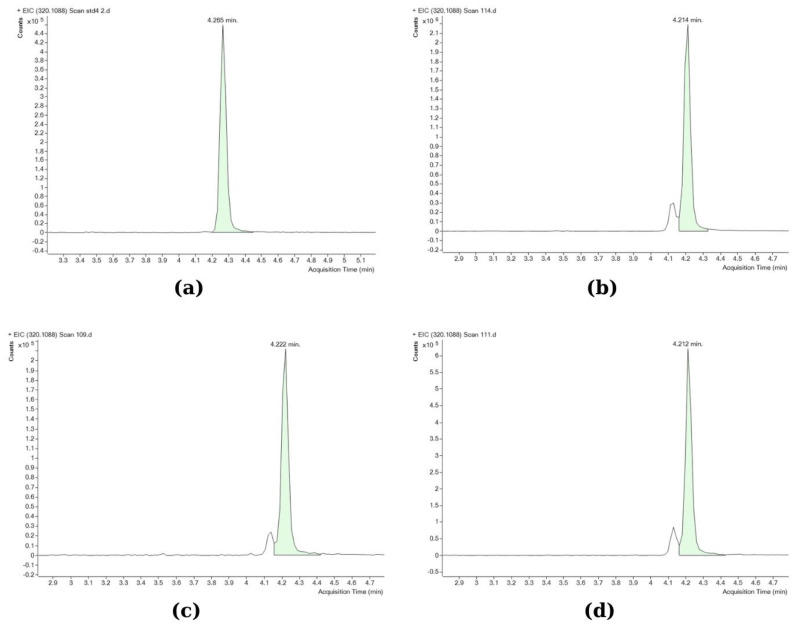
Q-TOF chromatograms of standard and different samples: (**a**) TTX standard (2 μg/mL); (**b**) gonad; (**c**) muscle; (**d**) liver (the smaller peak left to TTX in the tissue samples corresponds to 4-epiTTX, which was not included in the analysis). Accurate mass *m/z*: 320.1088; retention time window: 4200–4270 min; mass accuracy: 0.93 ppm; mass window: ± 5ppm.

TTX analyses were performed using an Agilent 6545 Accurate-Mass Q-TOF mass spectrometer coupled to an Agilent 1260 HPLC system (Agilent Technologies, Inc., Santa Clara, CA, USA). The Q-TOF LC/MS analysis was performed according to Kosker et al. [37]. A Poroshell 120 HILIC (3.0 × 50 mm; 2.7 µm) column (Agilent Technologies, Inc., Santa Clara, CA, USA) was used for the analyses. The toxin was separated in the column using two different mobile phases. Mobile phase A was 20 mM ammonium acetate in distilled water (Sigma-Aldrich), and mobile phase B was 20 mM ammonium acetate in acetonitrile (Sigma-Aldrich). The analysis was completed in 8 min, with the retention time of TTX being 4.2 min (Figure 2). A gradient program was used as follows: the first 2.5 min were set as 3% mobile phase A, 97% mobile phase B, then for 2 min 30% mobile phase A, 70% mobile phase B, and again between 4.5 and 8 min 3% mobile phase A, 97% mobile phase B. The column temperature was 20 °C, and the injection volume was 10 µL. The LC system was operated in the positive ion mode with the ESI (electrospray ionization) interface using the following parameters: drying gas flow, 10.0 L/min; nebulizer pressure, 40 psi; gas drying temperature, 325 °C; sheath gas temperature, 400 °C; sheath gas flow, and nitrogen at 12 L/min. The scanning was performed in the range of *m/z* 50–500. Only the parent TTX compound was determined, as other previous studies by Christidis et al. [39] and Bane et al. [60] have confirmed that it is the most abundant among all TTX analogues in *L. sceleratus* pufferfish, whereas the limited information on the toxic potential of individual TTX analogues, although probably present in the samples, would not allow for a solid evaluation of their contribution in overall toxicity. The Q-TOF LC/MS operating conditions were first optimized using the TTX standard. The method’s limit of detection (LOD; S/N > 3) was calculated at 0.008 μg/g and the limit of quantification (LOQ; S/N > 10) at 0.026 μg/g. The working mass range was from *m/z* 50 to 500 in full-scan acquisition mode. Quantitation was performed by using the MS mode; the accurate measured mass of the detected ion was [M + H]^+^, *m/z* 320.1088. The peaks were identified by retention time (4200–4270 min) and exact mass (mass window ±5 ppm, mass accuracy 0.93 ppm, monoisotopic pattern of the signals). Data processing was carried out using the Mass Hunter software. All Q-TOF LC/MS analyses were performed in triplicate.

### 3.5. Statistical Analysis

Results are reported as the mean and standard deviation of the measurements. SPSS version 17.0 (SPSS Inc., Chicago, IL USA) was used for the statistical evaluations of the monthly changes of TTX values among tissues and between males and females captured in the same month (Figure S1). To identify significant differences between monthly values of TTX levels in tissues, a one-way analysis of variance (ANOVA) combined with Duncan’s multiple range test comparisons at *p* < 0.05 were performed. The *t*-test was applied between different genders belonging to the same month.

## 4. Conclusions

The present study extends our knowledge on the TTX contents of *L. sceleratus* at monthly intervals and provides further data on toxin levels required for the establishment of more efficient risk management practices for both public health and the fishing industry around Mediterranean countries. Our results demonstrate that the Mediterranean *L. sceleratus* remains poisonous and unsafe for human consumption throughout the year. As such, authorities should carefully consider the risks posed by pufferfish, especially of this species, and carry out awareness activities to prevent their consumption under any circumstances. In addition, considering that pufferfish are the most important source of purified TTX to be used as a drug [61], it is possible that Antalya Bay could constitute an appropriate potential production site for TTX with the purpose of alleviating the detrimental effects of pufferfish expansion through overfishing. In this context, this study provides further insights into the months during which TTX levels are elevated in *L. sceleratus*. To summarize, the findings highlight the toxin levels of *L. sceleratus* in Antalya Bay by offering valuable information regarding the temporal distribution of TTX in this ecosystem. Further research on the monthly variation of TTX and its analogues could also be necessary to obtain more solid information on their individual contribution to the overall toxic burden of *L. sceleratus* and their occurrence patterns, as well as on TTX metabolism.

## Figures and Tables

**Table 1:** Monthly length and weight of pufferfish samples (*n* = 5, mean ± SD).

Months	Gender	Length (cm)	Weight (g)
February	♀	51.6 ± 2.96	1467.4 ± 253.49
	♂	49.5 ± 3.73	1309.8 ± 339.97
March	♀	52.9 ± 11.79	1702.7 ± 1058.14
	♂	52.5 ± 1.19	1665.4 ± 320.84
April	♀	54.5 ± 2.33	2062.2 ± 431.56
	♂	47.8 ± 1.63	1338.0 ± 249.61
May	♀	39.5 ± 1.95	750.9 ± 82.27
	♂	49.0 ± 6.07	1595.0 ± 539.36
June	♀	45.6 ± 1.45	1110.7 ± 110.86
	♂	40.9 ± 6.33	835.5 ± 396.92
July	♀	52.6 ± 2.99	1870.7 ± 472.26
	♂	41.1 ± 1.15	715.2 ± 331.30
August	♀	54.4 ± 4.48	1941.2 ± 305.86
	♂	44.2 ± 8.01	951.8 ± 326.46
September	♀	45.5 ± 5.69	1146.7 ± 484.61
	♂	47.3 ± 1.60	1243.3 ± 83.26
October	♀	53.0 ± 1.78	1664.1 ± 173.40
	♂	44.5 ± 3.90	1048.8 ± 281.14
November	♀	42.3 ± 0.46	844.9 ± 20.68
	♂	49.2 ± 0.44	1260.1 ± 57.30
December	♀	46.4 ± 3.26	1112.4 ± 323.96
	♂	49.4 ± 8.01	1403.99 ± 445.84
Overall (*n* = 110)	♀	47.8 ± 4.66	1316.0 ± 400.45
	♂	47.7 ± 4.60	1313.0 ± 399.49

**Table 2:** Monthly TTX levels (µg/g) in different tissues of *L. sceleratus* (mean ± SD).

Months	Gender	N	Muscle	Liver	Gonad
February	♀	5	2.8 ± 0.04 ^e,x^	8.1 ± 0.34 ^f,y^	21.0 ± 0.87 ^c,x^
	♂	5	1.3 ± 0.03 ^f,y^	21.6 ± 6.44 ^a,x^	20.2 ± 1.86 ^bc,x^
March	♀	5	1.5 ± 0.29 ^f,y^	0.8 ± 0.03 ^h,y^	6.8 ± 0.89 ^d,x^
	♂	5	3.6 ± 1.08 ^bc,x^	15.8 ± 4.37 ^b,x^	6.4 ± 0.85 ^de,x^
April	♀	5	5.6 ± 0.14 ^b,x^	13.7 ± 0.09 ^d,x^	25.0 ± 0.12 ^c,x^
	♂	5	3.8 ± 0.01 ^b,y^	7.6 ± 1.28 ^cd,y^	2.7 ± 0.00 ^e,y^
May	♀	5	3.5 ± 0.49 ^d,x^	7.4 ± 0.45 ^f,x^	16.2 ± 1.42 ^cd,x^
	♂	5	2.6 ± 0.06 ^de,y^	9.4 ± 1.26 ^cd,x^	1.4 ± 0.07 ^e,y^
June	♀	5	4.0 ± 0.36 ^d,x^	14.4 ± 1.42 ^cd,x^	8.6 ± 0.76 ^d,x^
	♂	5	3.7 ± 0.37 ^b,x^	9.2 ± 0.57 ^cd,y^	3.5 ± 0.06 ^e,y^
July	♀	5	2.5 ± 0.68 ^e,x^	11.1 ± 0.64 ^e,x^	7.5 ± 0.43 ^d,y^
	♂	5	2.9 ± 0.32 ^cd,x^	7.2 ± 1.09 ^cd,y^	25.9 ± 5.70 ^b,x^
August	♀	5	4. 8 ± 0.11 ^c,x^	16.8 ± 1.67 ^bc,x^	23.8 ± 0.80 ^c,x^
	♂	5	2.6 ± 0.22 ^de,y^	12.0 ± 1.15 ^bc,y^	19.3 ± 6.53 ^bc,x^
September	♀	5	3.7 ± 0.06 ^d,x^	14.9 ± 0.55 ^bcd,x^	38.2 ± 5.48 ^b,x^
	♂	5	2.8 ± 0.41 ^d,y^	11.8 ± 0.16 ^bc,y^	9.7 ± 1.27 ^de,y^
October	♀	5	1.5 ± 0.15 ^f,y^	3.5 ± 0.75 ^g,y^	9.0 ± 3.69 ^d,x^
	♂	5	1.9 ± 0.01 ^ef,x^	5.8 ± 0.52 ^d,x^	7.6 ± 1.20 ^de,y^
November	♀	5	5.5 ± 0.18 ^b,x^	17.4 ± 3.11 ^b,y^	65.9 ± 3.34 ^a,x^
	♂	5	5.9 ± 0.30 ^a,x^	25.3 ± 2.48 ^a,x^	68.2 ± 11.40 ^a,x^
December	♀	5	7.8 ± 0.07 ^a,x^	34.2 ± 2.63 ^a,x^	44.2 ± 15.61 ^b,x^
	♂	5	6.0 ± 0.37 ^a,y^	15.6 ± 1.22 ^b,y^	12.9 ± 0.21 ^cd,y^
Overall	♀	55	3.9 ± 1.92	12.9 ± 8.88	24.4 ± 19.13
	♂	55	3.4 ± 1.49	12.9 ± 6.19	15.9 ± 18.36

Different letters (a,b,c,d,e,f) in the same column represent the significant difference between TTX values in different months for each tissue (*p* < 0.05). Different letters (x,y) in the same column for each month show significant differences for both genders (*p* < 0.05). N: number of samples.

**Table 3:** TTX levels in different tissues of *L. sceleratus* from the Mediterranean (μg/g), reported using different liquid chromatography–mass spectrometry (LC-MS) methods.

Region	Muscle	Gonads	Liver	N	Analysis Method	Reference
Aegean Sea	<0.3–3.5	0.5–46.3	<0.3–44.2	43	LC-ESI-CID-MS/MS	Rodríguez et al. [36]
	ND	4.8	8.1	2	LC-MS/MS	Reverté et al. [33]
	0.5–2.1	NA	NA	2	LC-HRMS	Leonardo et al. [38]
	0.2–21.7	1.2–85.2	1.1–239.8	37	LC-MS/MS	Anastasiou et al. [50]
North–East Mediterranean	ND–2.8	0.4–52.1	ND–46.2	16	LC-MS/MS	Kosker et al. [42]
	0.7–5.1	0.7–35.6	0.9–21.1	20	Q-TOF LC-MS	Kosker et al. [2]
East Mediterranean	0.1–0.6	0.3–253.0	1.4–46. 7	3	LC-MS/MS	Hassoun et al. [40]
	0.1–3.4	0.2–80.0	0.1–25.4	20	LC-MS/MS	Acar et al. [43]
West Mediterranean	1.0	26.0	3.1	1	LC-MS/MS	Rambla-Alegre et al. [8]
	1.0	25.2	5.4	1	LC-HRMS	Rambla-Alegre et al. [8]
South Mediterranean	0.0–20.7	0.3–189.0	0.0–104.41	83	LC-MS/MS	Christidis et al. [39]
	7.6–36.5	2.1–1324.4	38.9–188.2	3	LC-MS/MS	Alkassar et al. [49]
Antalya Bay	1.3–7.8	1.4–68.2	0.8–34.2	110	Q-TOF LC-MS	Present Study

N, Number of individuals analyzed. LC-MS/MS, Liquid Chromatography with tandem mass spectrometry. LC-HRMS, Liquid chromatography–high-resolution mass spectrometry. LC-ESI-CID-MS/MS, liquid chromatography coupled to electrospray ionization mass spectrometry operating in the conventional mode in addition to low-energy collision dissociation tandem mass spectrometry. Q-TOF LC-MS, liquid chromatography quadrupole time-of-flight mass spectrometry. ND, not detected. NA, not Analyzed.

## Data Availability

The data presented in this study are available on request from the corresponding authors.

## Supplementary Materials

The following supporting information can be downloaded at: https://www.mdpi.com/article/10.3390/md21100527/s1, Figure S1: TTX spectra of different pufferfish samples.
