# Supplementary material for: Monthly Variation of Tetrodotoxin Levels in Pufferfish (Lagocephalus sceleratus) Caught from Antalya Bay, Mediterranean Sea
Source: Mar Drugs. 2023 Oct 5;21(10):527. doi: 10.3390/md21100527 (PMC10608123; doi:10.3390/md21100527)
Supplement: Supplementary file 1 [file marinedrugs-21-00527-s001.zip › marinedrugs-2581326-supplementary.pdf]

Article

Monthly variation of Tetrodotoxin levels in pufferfish (*Lagocephalus sceleratus*) caught from Antalya Bay, Mediterranean Sea

Supplementary material

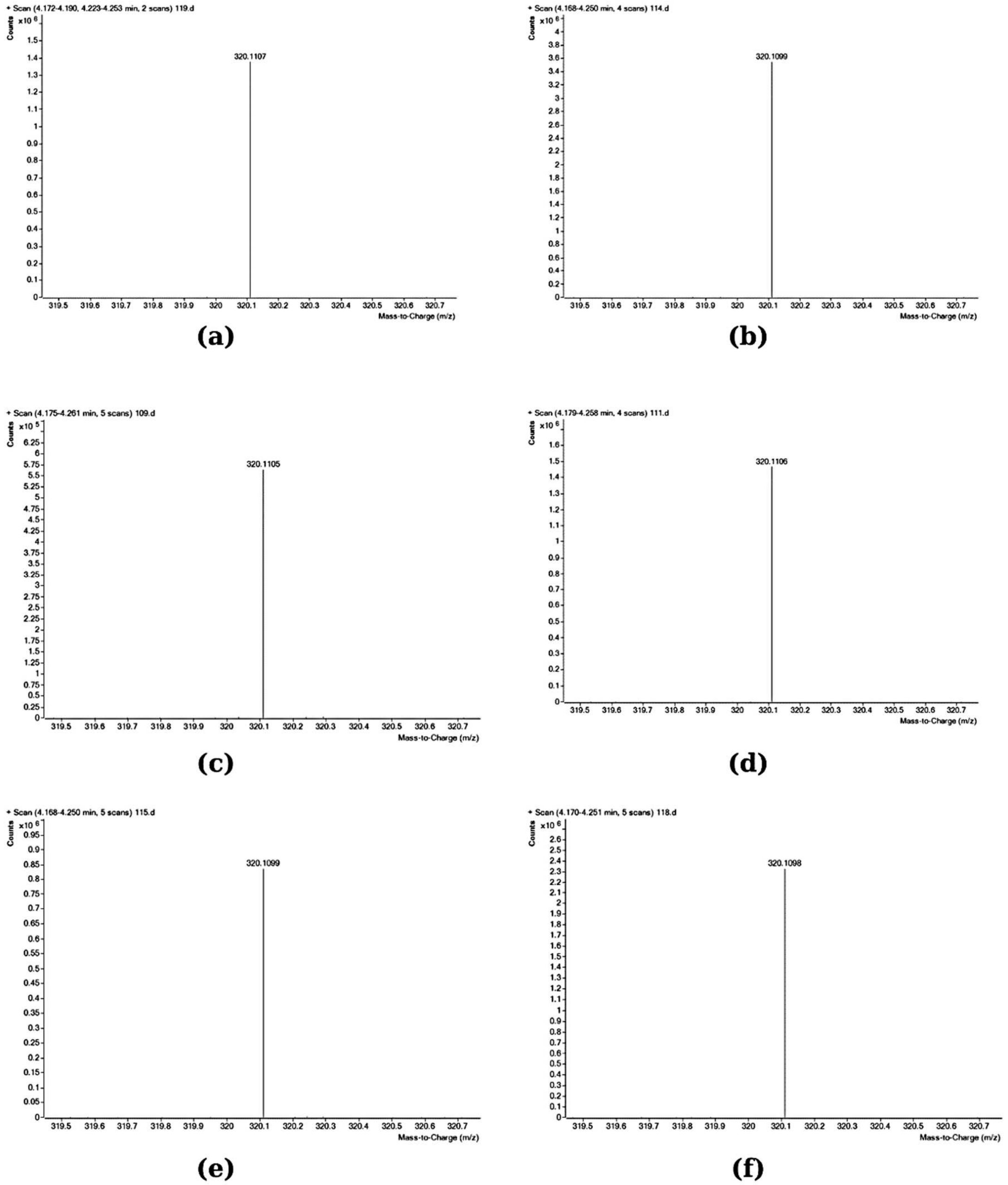

Figure S1: TTX spectra of different pufferfish samples: (**a**) male, gonad; (**b**) female, gonad; (**c**) female, muscle; (**d**) female, liver; (**e**) male muscle; (**f**) male, liver
